# A PPARα Promoter Variant Impairs ERR-Dependent Transactivation and Decreases Mortality after Acute Coronary Ischemia in Patients with Diabetes

**DOI:** 10.1371/journal.pone.0012584

**Published:** 2010-09-03

**Authors:** Sharon Cresci, Janice M. Huss, Amber L. Beitelshees, Philip G. Jones, Matt R. Minton, Gerald W. Dorn, Daniel P. Kelly, John A. Spertus, Howard L. McLeod

**Affiliations:** 1 Department of Medicine, Washington University School of Medicine, Saint Louis, Missouri, United States of America; 2 City of Hope National Medical Center, Duarte, California, United States of America; 3 University of Maryland, Baltimore, Maryland, United States of America; 4 Saint Luke's Mid America Heart Institute and the University of Missouri-Kansas City, Kansas City, Missouri, United States of America; 5 Burnham Institute for Medical Research, Orlando, Florida, United States of America; 6 University of North Carolina Institute for Pharmacogenomics and Individualized Therapy, Chapel Hill, North Carolina, United States of America; Harvard Medical School, United States of America

## Abstract

Activation of peroxisome proliferator-activated receptor alpha (PPARα) occurs in animal models of diabetes (DM) and is implicated in pathological responses to myocardial ischemia. Using bioinformatics, we identified a single nucleotide polymorphism (SNP) in the PPARα gene promoter (*PPARA* −54,642 G>A; rs135561) that altered the consensus sequence for a nuclear receptor binding site. Electrophoretic mobility shift assays showed that the domain bound two known *PPARA* transcriptional activators, estrogen-related receptor (ERR)-α and -γ and that *PPARA* G bound with greater affinity than *PPARA* A (>2-fold; P<0.05). Likewise, promoter-reporter analyses showed enhanced transcriptional activity for *PPARA* G vs. *PPARA* A for both ERR-α and -γ (3.1 vs.1.9-fold; P<0.05). Since PPARα activation impairs post-ischemic cardiac function in experimental models of DM, we tested whether decreased *PPARA* transcription in *PPARA* A carriers favorably impacted outcome after acute coronary ischemia in 705 patients hospitalized with acute coronary syndromes (ACS; 552 Caucasian, 106 African American). *PPARA* A allele frequencies were similar to non-diseased subjects. However, *PPARA* genotype correlated with 5-year mortality in diabetic (22.2% AA vs. 18.8% AG vs. 39.5% GG; P = 0.008), but not non-diabetic (P = 0.96) subjects (genotype by diabetes interaction P = 0.008). In the diabetic ACS subjects, *PPARA* A carriers had strikingly reduced all-cause mortality compared to *PPARA* G homozygotes, (unadjusted HR 0.44, 95% CI 0.26–0.75; P = 0.003; adjusted HR 0.48, 95% CI 0.27–0.83; P = 0.009). Consistent with previous descriptions of PPARα in experimental models and human disease, we describe a novel *PPARA* promoter SNP that decreases transcriptional activation of *PPARA* and protects against mortality in diabetic patients after ACS.

## Introduction

Peroxisome proliferator-activated receptor alpha (PPARα) is a transcription factor involved in the regulated expression of genes that control fatty acid uptake and metabolism. PPARα is highly expressed in the heart and other tissues that rely on fatty acid oxidation as their primary energy substrate [1]. Under normal conditions, regulation of myocardial PPARα expression and activity contributes to maintaining homeostatic balance between cellular fatty acid and glucose utilization via PPARα-mediated activation of target genes. This balance is perturbed by acute coronary ischemia [2]–[11], where myocardial PPARα expression can be a powerful determinant of functional recovery. For example, studies in which PPARα gene expression was experimentally manipulated in mouse models have demonstrated that forced myocardial PPARα overexpression decreases cardiac recovery after ischemia-reperfusion injury [12], whereas lowering PPARα expression protects against ischemic damage [12], [13]. Accordingly, genetic and epigenetic factors that influence PPARα gene (*PPARA*) expression have the potential to modify outcomes following acute coronary ischemia in patients.

Relevant to the human condition, we and others have suggested that the pathophysiological impact of PPARα may be greater in diabetic heart disease [7], [8], [14]. Insulin resistance and diabetes mellitus cause a shift of myocardial energy metabolism away from glucose and toward increased reliance on fatty acids that is driven in part by activation of PPARα [4], [15]. Animal models of diabetes and obesity, including STZ-induced diabetic rats, db/db mice, and ob/ob mice, show increased cardiac PPARα activation [15]–[17]. Given that cardiac PPARα is activated in DM, such a cardiac disease-modifier role might have special importance in diabetic patients. Indeed, a previously described single nucleotide polymorphism (SNP) within intron 1 of *PPARA* has been associated with the age of onset, and progression to insulin monotherapy, in patients with type 2 DM [18], although the biological mechanism for this effect has not been elucidated. Here, we identify and characterize a pair of linked SNPs in the 5′-flanking promoter region of human *PPARA* that modify a consensus nuclear hormone receptor response element to decrease *PPARA* transcription. We found that this genotype confers protection against early post-ischemic mortality that is specific to diabetic patients. Since these SNPs are also in linkage disequilibrium (LD) with the previously described intronic polymorphism, these results also suggest a mechanism for the clinical effects of that SNP.

## Methods

### Ethics Statement

The study was approved by the University of Missouri Kansas City Adult Health Sciences Institutional Review Board, the Saint Luke's Hospital of Kansas City Institutional Review Board and the Washington University Human Research Protection Office Institutional Review Board. Written informed consent was provided by each participant. A separate consent form for the acquisition of blood for genetic analysis was signed by each participant.

### Subjects

Patients were prospectively enrolled into the INFORM ACS registry at two Kansas City hospitals as previously described [19], [20]. Between 3/1/01 and 10/31/02, 1199 patients met the criteria for ACS using standard, accepted definitions of MI (n = 680) and unstable angina (n = 519) [21], [22]. MI patients were defined by an elevated troponin blood test [21]. Patients with DM were defined by having been given a diagnosis of DM by the referring physician and/or being treated with oral hypoglycemic agents or insulin. Three physicians reviewed the charts of all patients with diagnostic uncertainty and attained consensus on the final diagnosis.

Although there were no differences in gender (93.2% of men vs. 92.1% of women), Caucasians (91.5% vs. 98.3%, p<0.001) and older patients (mean age for those consenting = 61±13 vs. 65±13 for those not consenting, p = 0.004) were less likely to consent to DNA testing. 726 patients were enrolled in the genetic portion of this registry, constituting the cohort for the present analysis.

### Mortality Assessment

The Social Security Administration Death Master File was queried to determine patients' vital status as of 03/12/2008 (http://www.ntis.gov/products/ssa-dmf.asp).

### Bioinformatic Analysis

As an initial approach to discovering functionally significant polymorphisms of the *PPARA* promoters, we used the web-based program PromoLign (http://polly.wustl.edu/promolign/main.html) [23], to identify nucleotide variants within 10 kb of the transcription start site. This program identifies promoter polymorphisms that are within human-mouse homologous blocks and/or occur within, and alter, the consensus sequences for putative trans-activating factor binding sites (output shown in Supplemental Figure S1). We considered that four essential criteria were necessary to assign functional importance to *PPARA* promoter polymorphisms: 1. The bioinformatic SNP had to be validated in a multi-ethnic human cohort; 2. The putative nuclear receptor binding domain in which it is located had to bind a transcription factor; 3.The SNP had to alter binding of that transcription factor; and 4. The SNP had to change gene transcriptional activity.

### Targeted Resequencing

Targeted resequencing of the putative nuclear receptor domain was performed using pyrosequencing in 380 DNA samples from unrelated, random, healthy blood donors, 95 each from Caucasians, African-Americans, Han Chinese, and Mexicans (Coriell Institute for Medical Research, Camden NJ).

### Genotyping

DNA was isolated and extracted using the Puregene genomic DNA purification kit (Gentra, Minneapolis, MN). The DNA segments containing the region of interest were amplified with the polymerase chain reaction (PCR). PCR primers were designed using Primer3 online software (http://fokker.wi.mit.edu/cgi-bin/primer3/primer3_www.cgi) [24], and pyrosequencing primers were designed using the Pyrosequencing SNP Primer Design Version 1.01 software (http://www.pyrosequencing.com). Before use, PCR primer sequences were screened across the human genome using the NCBI Blast program to ensure their specificity for the gene of interest. PCR was carried out using Amplitaq Gold PCR master mix (ABI, Foster City, CA), 1 pmole of each primer (IDT, Coralville, IA), and 1ng DNA. The PCR primers and conditions are listed in Supplemental Table S1. Pyrosequencing was performed using the PSQ HS 96A system with MA v2.0 software as previously described [25]. Data were automatically transferred from the PSQ HS 96A to a Microsoft Access database for permanent storage and merging with the clinical datasets through SAS v9.1. Pair wise linkage (D′) and haplotype analysis was carried out using the Polymorphism and Haplotype Analysis Suite [26].

### *In vitro* Studies

Electrophoretic mobility shift assays used standard methodologies that have been previously described [27], [28]. Complementary oligonucleotides corresponding to the *PPARA* promoter region encompassing the SNP of interest were annealed to generate double-stranded fragments for radiolabeling or cloning. Oligonucleotide sequences were as follows: *PPARA* −54,642 G forward: gatcTAGCTCCTGCAGGTTCTCAAGGTTGTAG**C** CC**G**CACCCTGCT

*PPARA* −54,642 A forward: gatcTAGCTCCTGCAGGTTCTCAAGGTTGTAG**T** CC**A**CACCCTGCT. Note that these sequences differed at two sites, the −54,642 SNP site and at a site 3 nucleotides upstream of this SNP site, due to the upstream site being in complete LD with the −54,642 SNP site; see results. The positive control element consisted of the previously characterized estrogen-related receptor (ERR) responsive element [28]. Probes were synthesized by Klenow fill-in reaction with [γ-^32^P]dCTP using the double-stranded fragments and used at 15,000 cpm per reaction. Binding reactions were performed as described [28] using recombinant ERRα and ERRγ generated from TNT Quick Coupled T7 reticulocyte lysate (Promega). In competition assays cold competitor was added at the indicated concentrations simultaneously with probe and protein. Gels were imaged on a Storm phosphorimager and band intensities were quantified using ImageQuant software (Molecular Dynamics).

To clone heterologous *PPARA* variant promoter-reporter constructs, (−54,642A)2c.TK.Luc and (−54,642G)2c.TK.Luc, double-stranded fragments corresponding to the probes used in EMSA were 5′ phosphorylated then ligated into the BamHI site of a luciferase expression vector immediately upstream of the thymidine kinase minimal promoter of the pGL2.TK.Luc reporter plasmid. Clones were screened by PCR and those carrying 2 element copies were verified by sequence analysis. Transient transfection studies were performed as previously described [28], [29]. *PPARA* promoter-reporter constructs were cotransfected with empty expression vector or vector expressing ERRα or ERRγ. Transient transfections in CV1 cells were performed using Lipofectamine 2000 (Invitrogen) with 2.7 µg/ml reporter, 0.3 µg/ml each expression vector, and 0.3 µg/ml pRL-CMV to control for transfection efficiency. C2C12 myoblasts were transfected with 4 µg/ml reporter, 0.5 µg/ml each expression vector, and 0.5 µg/ml pRL-CMV using the calcium phosphate precipitation method. Firefly luciferase activity normalized to that of Renilla luciferase was measured 48h post-transfection on a Clarity luminescence microplate reader (BioTek) with triplicate determinations in four trials.

### Statistical Analyses

Baseline patient characteristics were summarized and compared stratified by genotype. Hardy-Weinberg equilibrium was assessed by chi-square in Caucasians and African Americans separately. Continuous variables were reported as mean ± standard deviation and compared using *t*-tests. Lipid values had skewed distributions, were log-transformed prior to analysis, and were summarized by median and interquartile range. Categorical variables were summarized by frequency and percent and compared using chi-square or Fisher's exact tests, when the expected cell size was <5.

The primary outcome was time to all-cause mortality through 60 months. Event rates by *PPARA* −54,642 genotype were calculated using Kaplan-Meier analysis and compared using log-rank tests. The relative hazard associated with each genotype was estimated using Cox proportional hazard models, both in crude analysis and adjusting for demographics (age, gender, race) and characteristics that were significantly different across genotypes (systolic BP and treatment strategy).

Proportional hazard assumptions were verified using Schoenfeld residuals. P-values<0.05 were considered statistically significant. Analyses were performed using SAS version 9.1 (SAS Institute, Inc., Cary, NC) and R version 2.2.0.(http://www.R-project.org).

Luciferase activity and binding level values were summarized by mean and standard error and compared using *t*-tests. P values<0.05 were considered statistically significant.

## Results

### Bioinformatics analysis of *PPARA* promoters A–D

The PPARα gene (*PPARA*) has a complex 5′ structure, consisting of four distinct promoter regions designated promoters A–D [30]. 13 nucleotide variants were identified within 10 kb of the transcription start site as described in Methods (Table 1 and Supplemental Figure S1). Of the 13 *PPARA* promoter polymorphisms in the database within human-mouse homologous blocks, only 2 single nucleotide polymorphisms (SNPs) located 3 nucleotides apart from one another, occurred within, and altered the consensus sequences for, putative trans-activating factor binding sites. Of particular interest because of the previously established important effects of nuclear hormone receptors on *PPARA* expression [28], [31], was a G to A substitution located 6,862 nucleotides upstream of the major *PPARA* transcription start site of promoter A that disrupts a nuclear receptor half-site, designated *PPARA* −54,642 G>A (based on its position relative to the translation initiation site; rs135561). This domain is highly conserved across vertebrate species, including mouse, rat, rabbit, dog, chicken, elephant and human (Supplemental Figure S2) and was therefore selected for further genotyping and analysis.

**Table 1:** 13 nucleotide variants identified within 10 kb of the PPARA transcription start site located within human-mouse homologous blocks and/or within consensus sequences for putative trans-activating factor binding sites.

SNP	Allele	Chromosome 22 Position	TransFac factor binding site(s) in human sequence around this SNP
rs4823568	A/T	46537906	No
rs135562	A/G	46539636	Yes
rs135561	C/T	46539639	Yes
rs135559	A/G	46539706	No
rs135558	A/−	46540237	No
rs135557	C/T	46541227	No
rs6007947	C/T	46541429	No
rs3052727	−/AGA	46542331	No
rs4044314	A/G	46542403	No
rs2018221	C/T	46542455	No
rs717926	A/C	46542597	No
rs135556	A/G	46543485	No
rs4253781	C/T	46547379	No

### The *PPARA* −54,642 A variant is in complete LD with a *PPARA* −54,645 T variant and is the major allele in African Americans

Targeted resequencing of the putative nuclear receptor domain was performed using pyrosequencing in 380 DNA samples from random, healthy blood donors, 95 each from Caucasians, African-Americans, Han Chinese, and Mexicans (Coriell Institute for Medical Research, Camden NJ). The *PPARA* −54,642 G>A substitution was confirmed, as was a C to T substitution three nucleotides 5′ (*PPARA* −54,645 C>T; rs135562) that was in complete LD (D′ = 1, r^2^ = 93, P<0.001) in all ethnic cohorts. Frequencies of the variant *PPARA* −54,642 A/*PPARA* −54,645 T allele were 0.27 in the Caucasian cohort, 0.68 in the African-American cohort, 0.15 in the Chinese cohort, and 0.12 in the Mexican cohort, and were each consistent with predictions of Hardy-Weinberg equilibrium. Thus, the *PPARA* −54,642 A variant (and its linked upstream partner) is relatively common in Caucasians, and is the most common allele in African Americans, but is relatively uncommon among Mexicans and Asians (≤15%).

### The *PPARA* −54,642 G>A SNP alters ERRα and ERRγ binding and *PPARA* transcriptional activation

**Figure 1:**
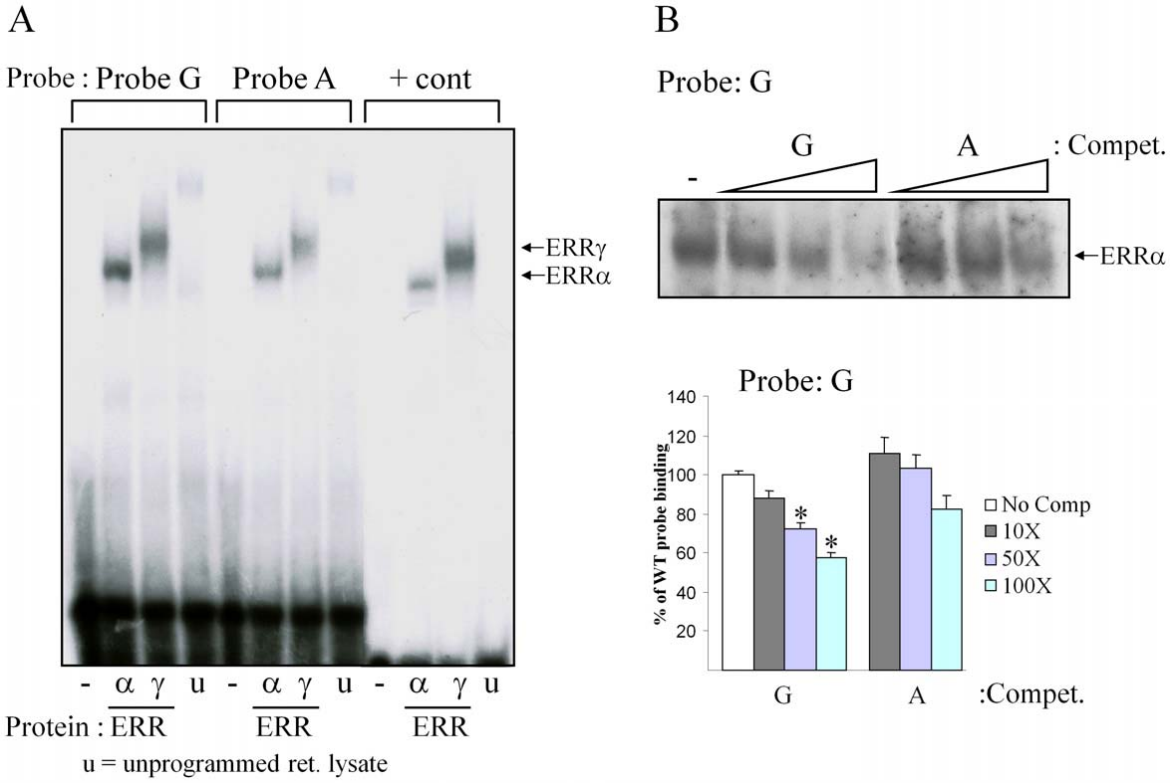
*PPARA* −54,642 A variant binds ERRα and ERRγ with less affinity. (**A**) Electrophorectic mobility shift assay of binding activity performed with ^32^P-labeled probes differing at the *PPARA* −54,642 SNP sites (with the corresponding linked −54,645 SNP; see methods) using recombinant ERRα and ERRγ proteins. (**B**) Competition experiments performed with *PPARA* −54,642 G probe incubated with 10-fold, 50-fold and 100-fold excess of unlabeled G probe or unlabeled A probe as a competitor. Mean relative band intensities (representative trial, **top**) from 3 trials were quantified by phosphorimage analysis and results are depicted graphically in the **bottom** panel. Asterisks represent significantly different binding to probe compared to control (p<0.05).

Having confirmed the bioinformatics in a multi-racial human cohort, the next step was to determine if there were any effects of the *PPARA* promoter SNP on transcription factor binding and transcriptional activity. We considered that the most likely factors to bind to the *PPARA* nuclear receptor half-site were estrogen related receptor (ERR)α and ERRγ, based on the characteristic nucleotide sequence [32], [33], on known similarities in tissue expression between ERR and PPARα [34]–[36], and on ERR regulatory activities for genes downstream of PPARα [28], [35], [36]. To test ERR binding, a 49 bp double-stranded (ds) oligonucleotide was generated corresponding to the ‘wild-type’ form of *PPARA* −54,642 (G) and −54,645 (C). Electrophoretic mobility shift assays (EMSA) were performed to compare binding of recombinant ERRα and ERRγ proteins between ^32^P-labeled *PPARA* and authentic, previously described ERR binding sequences [28]. Both ERRα and ERRγ bound the wild-type *PPARA* −54,642 G sequence with activities equal to, or greater than, the control ERR responsive element (Probe G vs. +control; Figure 1A).

We next examined relative ERR binding in EMSA studies performed with the same wild-type *PPARA* −54,642 G probe described above and a ‘variant’ *PPARA* −54,642 A probe, identical in sequence except for the nucleotides encoding the two linked SNPs (A at nucleotide −54,642 and T at nucleotide −54,645, respectively). The apparent binding of both ERRα and ERRγ for the variant *PPARA* −54,642 A probe was qualitatively less than for the wild-type G probe (Probe A vs. Probe G; Figure 1A). The relative affinity of ERRα for the wild-type and variant sequences was assessed in competition binding studies using increasing amounts of unlabeled G or A dsDNA probe to displace wild-type G probe in the EMSA binding reactions. The variant A probe competed less effectively for binding to the ^32^P-labelled wild-type sequence (IC_50_ of A probe 2.3-fold greater than G probe, P<0.05), demonstrating a lower affinity for ERRα (Figure 1B and 1C) and ERRγ (data not shown). Thus, the G>A substitution decreases *PPARA* promoter affinity for the transcription factors ERRα and ERRγ.

**Figure 2:**
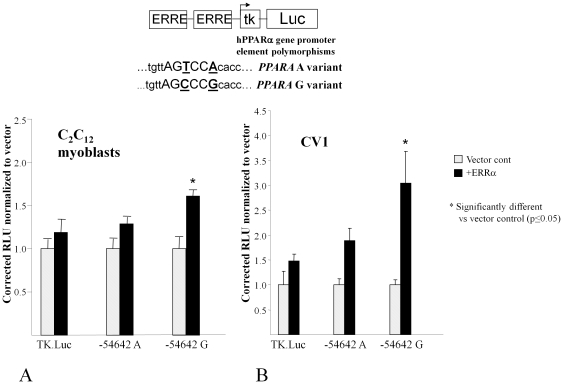
*PPARA* −54,642 A variant is less responsive to ERR-mediated co-activation. Mean normalized luciferase activities (±SE) in (**A**) C2C12 myobalst cells or (**B**) CV1 cells cotransfected with *PPARA* −54,642 A or *PPARA* −54,642 G promoter-reporter constructs +/− a mammalian expression vector that overexpresses ERRα as indicated. Asterisks represent significantly different transcriptional activation compared to vector control (p<0.05). Four independent trials were performed in triplicate for each cell line.

To determine whether the decrease in ERR binding afforded by the *PPARA* −54,642 G>A polymorphism altered transcriptional activity, we performed reporter assays. The reporter constructs contained two copies of either the wild-type G or the variant A 49 bp promoter element inserted upstream of a minimal thymidine kinase (TK) promoter-luciferase reporter [29]. When co-transfected with an ERRα expression plasmid into C2C12 myoblasts, which are a useful *in vitro* model of skeletal and cardiac muscle [37], the variant A promoter-reporter was ∼25% less responsive to ERRα-mediated activation than the wild-type G promoter-reporter (p<0.05; n = 4; Figure 2A). Similar decreased activation of variant *PPARA* −54,642 A was observed with ERRγ co-transfection (data not shown).

To control for the possibility that endogenous ERRα in C2C12 myoblasts was masking *PPARA*/TK A promoter-reporter activation by transfected ERRα, we repeated the experiments in CV1 cells that are null for endogenous ERRα and ERRγ [28]. In the CV1 system the wild-type G *PPARA* promoter-reporter had ∼60% greater responsiveness to ERRα-mediated activation than the A variant promoter-reporter (3.1-fold vs.1.9-fold; P<0.05; n = 4, Figure 2B). Again, the results were similar with co-transfection of ERRγ (data not shown). These studies demonstrate that the variant *PPARA* promoter sequence exhibits diminished ERR binding and decreased ERR-mediated transcriptional activation, compared to the wild-type *PPARA* −54,642 G allele.

### In diabetic patients, *PPARA* -54,642 A allele carriers have deceased mortality after acute coronary ischemia

The above studies demonstrate clear biological effects of the *PPARA* −54,642 G>A promoter polymorphism: The more common (in Caucasians) *PPARA* G promoter is more responsive to ERR, while the variant A promoter is less effective at binding ERR and is therefore resistant to ERR-mediated transcription. Since we have previously shown that cardiac PPARα is activated in experimental DM [15], and that such PPARα activation is deleterious during experimental myocardial ischemia [38]–[49], we hypothesized that diminished PPARα gene activity conferred by the −54,642 A variant might have the opposite effect, i.e. be protective, in human subjects with acute coronary ischemic syndromes (ACS). We tested this idea in the 726 subject genetic sub-study of the INFORM trial of outcomes after ACS [19]. Baseline clinical and demographic data for the 705 patients (96%) for whom genotypes were obtained are listed in Table 2, and for patients with DM stratified by *PPARA* promoter genotype in Table 3. The *PPARA* −54,642 A allele frequency in ACS patients was 0.30 in Caucasians and 0.60 in African-Americans, which is not significantly different from the frequencies in the healthy volunteers used for SNP validation (P = 0.53 and  = 0.26, respectively; see above), and suggests that the *PPARA* SNP is not an independent risk factor for cardiac ischemic disease. As in the non-diseased cohort, the adjacent *PPARA* −54,645 C>T SNP (rs135562) was found to be in complete LD (D′ = 1, r^2^ = 93, P<0.001) in both Caucasian and African-American subgroups, and did not deviate significantly from Hardy-Weinberg equilibrium.

**Table 2:** Clinical Characteristics of the INFORM ACS Cohort.

Patient Characteristics	n = 705
Age	60.6±12.5
Sex	
Male	449 (63.7%)
Female	256 (36.3%)
Race	
Caucasian	552 (78.3%)
African American	126 (17.9%)
Other	27 (3.8%)
Prior MI	236 (33.5%)
Prior PCI	221 (31.3%)
Prior CABG	128 (18.2%)
Chronic HF	55 (7.8%)
HTN	464 (65.8%)
Hyperlipidemia	427 (60.6%)
DM Known on Arrival	200 (28.4%)
Admit BMI	29.6±6.3
Admit systolic BP	137.1±26.9
EF	47.2±12.9
Admit Glucose	148.2±83.0
ACS Type	
STEMI	201 (28.5%)
NSTEMI	215 (30.5%)
USA	289 (41.0%)
Cardiac catheterization	576 (81.7%)
Treatment	
Medical management	264 (37.4%)
PCI	409 (58.0%)
CABG	32 (4.5%)
Discharge Medications - BB	566 (80.5%)

**Abbreviations**: MI myocardial infarction, PCI percutaneous coronary intervention, CABG coronary artery bypass graft, HF heart failure, HTN hypertension, DM diabetes mellitus, BMI body-mass index, BP blood pressure, EF ejection fraction, ACS acute coronary syndrome, STEMI ST-elevation myocardial infarction, NSTEMI non-ST-elevation myocardial infarction, USA unstable angina, BB beta-blockers.

**Table 3:** Clinical Characteristics of the Diabetic INFORM ACS Cohort according to *PPARA* −54,642 genotype.

	*PPARA* −54,642 AG			
Patient Characteristics by Genotype	AA (n = 36)	AG (n = 75)	GG (n = 89)	p-values
Age (mean +/− SD)	57.4+/−11.0	59.1+/−11.5	61.4+/−10.3	0.132
Sex				0.659
Male	22 (61.1%)	39 (52.0%)	48 (53.9%)	
Female	14 (38.9%)	36 (48.0%)	41 (46.1%)	
Race				<0.001
Caucasian	16 (44.4%)	48 (64.0%)	72 (80.9%)	
African American	19 (52.8%)	26 (34.7%)	9 (10.1%)	
Other	1 (2.8%)	1 (1.3%)	8 (9.0%)	
HTN	29 (80.6%)	56 (74.7%)	79 (88.8%)	0.063
Admit BMI (mean +/− SD)	33.4+/−7.8	32.2+/−6.9	32.0+/−6.9	0.599
Admit systolic BP (mean +/− SD)	141.8+/−32.5	143.5+/−25.9	132.4+/−26.5	0.028
EF<40%	7 (20.0%)	18 (26.9%)	21 (26.3%)	0.724
Admit Glucose (mean +/− SD)	261.7+/−198.9	183.5+/−81.4	206.2+/−106.7	0.065
ACS Type				0.938
STEMI	8 (22.2%)	16 (21.3%)	19 (21.3%)	
NSTEMI	11 (30.6%)	20 (26.7%)	29 (32.6%)	
USA	17 (47.2%)	39 (52.0%)	41 (46.1%)	
Treatment				0.022
Medical management	22 (61.1%)	39 (52.0%)	34 (38.2%)	
PCI [acute or other]	11 (30.6%)	33 (44.0%)	53 (59.6%)	
CABG	3 (8.3%)	3 (4.0%)	2 (2.2%)	
Discharge Medications -BB	27 (75.0%)	63 (85.1%)	67 (75.3%)	0.252

**Abbreviations**: HTN hypertension, BMI body-mass index, BP blood pressure, EF ejection fraction, ACS acute coronary syndrome, STEMI ST-elevation myocardial infarction, NSTEMI non-ST-elevation myocardial infarction, USA unstable angina, PCI percutaneous coronary intervention, CABG coronary artery bypass graft, BB beta blocker.

**Figure 3:**
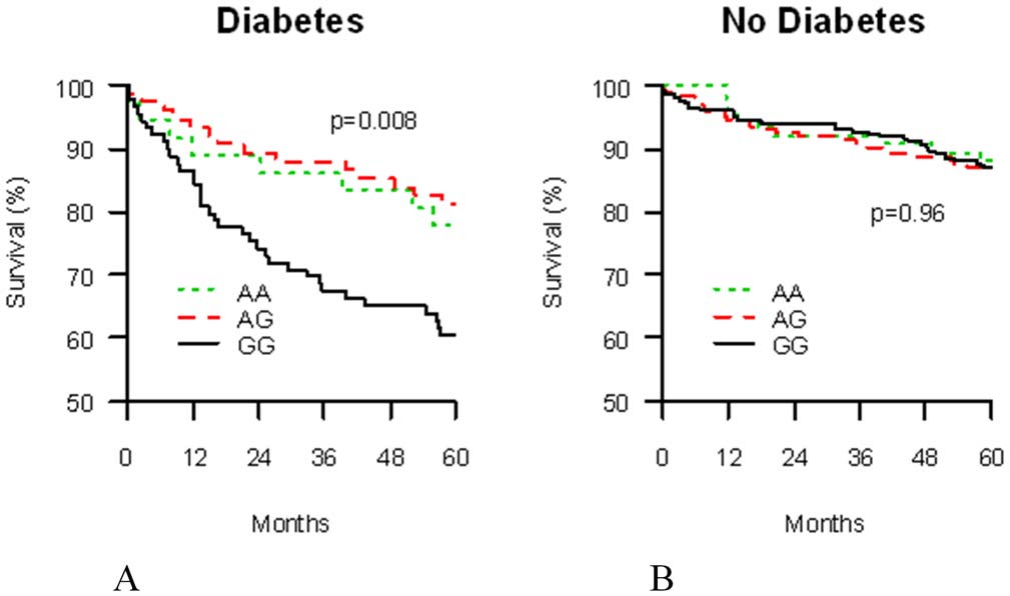
In diabetic patients, *PPARA* −54,642 A allele carriers have deceased mortality after acute coronary ischemia. Kaplan-Meier estimates of mortality stratified by *PPARA* −54,642 genotype in patients with (**A**) and without (**B**) DM. p-values between genotype groups are shown. (p-value for genotype by diabetes interaction = 0.008).

To determine if the *PPARA* promoter variant modified outcome in patients with acute coronary ischemic syndromes we examined the relationship between *PPARA* −54,642 (rs135561) genotype and five year mortality in the INFORM study. When DM status was not a co-variate, there was no apparent association. However, when the cohort was stratified by DM status, *PPARA* −54,642 genotype was associated with increased 5-year mortality in diabetic (p = 0.008) but not in non-diabetic (p = 0.96) ACS patients (Figure 3; genotype by DM interaction p = 0.008). In non-diabetic ACS patients, 5-year mortality was 13.5% for wild-type *PPARA* GG homozygous subjects, and 12.7% for variant *PPARA* A allele carriers (Figure 3B). Consistent with the known adverse effects on ACS outcome of having diabetes [50]–[52], homozygous −54,642 GG (wild-type) *PPARA* ACS patients had a 5-year mortality rate of 39.5% (Figure 3A). By contrast, *PPARA* variant A allele carriers (AG or AA) had a 5-year mortality rate following admission for ACS of 19.9% (Figure 3A and Table 4; HR 0.44, 95% CI 0.26–0.75; P = 0.003), demonstrating a striking protective effect of the *PPARA* promoter polymorphism. When adjusted for age, race, and gender, genotype remained an independent predictor of 5-year mortality, with *PPARA* −54,642 A allele carriers having less than half the relative risk of death within 5 years of presenting with ACS, compared to wild-type (homozygous *PPARA* −54,642 GG genotype) diabetics (HR 0.48, 95% CI 0.27–0.83; p = 0.009). This relationship was not changed by further adjusting for any other variable that differed significantly between genotype groups (HR 0.48, 95% CI 0.26–0.89; p = 0.019). Notwithstanding the differences in *PPARA* −54,642 allele frequency between Caucasians and African-Americans, in Caucasian DM subjects alone (the largest racial group) *PPARA* −54,642 A carriers were significantly protected against 5-year mortality compared to wild-type GG allele patients (unadjusted HR 0.47, 95% CI 0.24–0.90; P = 0.023; adjusted HR 0.46, 95% CI 0.24–0.89; P = 0.021; Table 4).

**Table 4:** Unadjusted and adjusted 5-year mortality for *PPARA* −54,642 A allele carriers.

	5-year Mortality	HR (*PPARA* −54,642 A-carriers)	95% CI	p-value
**Entire INFORM Cohort with DM**	unadjusted	0.44	0.26–0.75	0.003
	adjusted for age, race and gender	0.48	0.27–0.83	0.009
**Caucasian INFORM with DM**	unadjusted	0.47	0.24–0.90	0.023
	adjusted for age, race and gender	0.46	0.24–0.89	0.021

As mentioned above, an A>C SNP (rs135539) located in intron 1 (promoter B) of *PPARA*, 35,014 base pairs upstream of the translation start site, has been reported to influence the age of onset and progression to insulin monotherapy in patients with type 2 DM [18]. Although the authors provided no mechanism to explain these findings, they speculated that C allele carriers had reduced PPARα expression [18]. To evaluate whether *PPARA* −35,014 genotype impacted our findings, we genotyped the 726 subject genetic substudy of the INFORM trial for this polymorphism and found that the two SNPs (*PPARA* −54,642 and *PPARA* −35,014) were in moderate LD (D′ = 0.58, r^2^ = 17, P<0.001 Caucasians; D′ = 0.78, r^2^ = 39, P<0.001 African Americans). In addition, we found a borderline significant association with mortality in diabetic subjects within our cohort (unadjusted P = 0.051; AA vs. AC vs CC). Although this association only achieved borderline significance, there was a highly significant genotype by diabetes interaction (p = 0.009).

## Discussion

The current results define a new genetic disease modifier of outcome after cardiac ischemia that is specific for diabetes and suggests a biologic mechanism for the previously described *PPARA* intronic SNP. We used bioinformatics to identify candidate SNPs with the potential to modify transcription factor binding and high homology to mouse promoter regions in the four *PPARA* promoters. One candidate, the *PPARA* −54,642 (rs135561) G>A polymorphism, interrupted a putative nuclear factor biding domain and altered binding of two critical regulatory factors for *PPARA* expression, ERRα and ERRγ. *In vitro* promoter-reporter assays revealed that this effect changed transcriptional activity of the *PPARA* promoter, and our clinical association studies revealed a significant impact on 5-year mortality in patients with DM and acute coronary ischemia.

We took advantage of an experiment of nature, a common pair of SNPs that alter *PPARA* promoter activity, to demonstrate that *PPARA* expression level helps determine outcome after myocardial infarction and unstable angina in human subjects with DM. This result is consistent with previous findings in genetic mouse models that PPARα expression level inversely correlates with post-ischemic cardiac function [12] and extends the previous report of an association between *PPARA* −35,014 (rs135539) and onset and progression of DM [18]. Taken together, the human, mouse, and cell culture studies represent a comprehensive evaluation of PPARα effects in ischemic hearts.

A growing body of evidence describes associations between the PPARα gene and cardiovascular disease, risk or outcomes in subjects with type 2 DM [18], [53]–[58]. Of particular relevance to the current findings is the A>C SNP (rs135539) located in intron 1 of *PPARA* reported to influence the age of onset and progression to insulin monotherapy in patients with type 2 DM [18]. No mechanism has been forthcoming to explain these findings but the authors speculated that C allele carriers had reduced PPARα expression [18]. The current results suggest an alternate explanation for the previously described associations with the *PPARA* intronic SNP and provide evidence to support the authors' hypothesis that C allele carriers have reduced PPARα expression.

Increased risk of mortality after ACS in patients with DM has been recognized for many years [38]–[41], [43], [45], but the specific reasons for this adverse outcome are unknown. It is notable that the recent therapeutic advances that have improved general outcomes in ACS, have not impacted the disparity in outcomes in diabetic individuals [42], [44], [46]–[49]. Data from clinical trials also suggest that PPAR activation with pharmacological agonists increases the risk of myocardial infarction and adverse cardiovascular events in patients with DM [59], [60]. Since our results identify a gene-disease interaction that alters mortality specifically in the diabetic population after cardiac ischemia, they suggest that a therapeutic approach targeting PPAR-responsive genes and metabolic pathways might favorably affect outcomes.

Our studies suggest that modified ERR binding is a mechanism for altered *PPARA* expression in the polymorphic promoter, but there is scant available data on factors that affect ERRα and ERRγ mRNA or protein expression levels *in vivo*. We previously found that ERRα and ERRγ activate *PPARA* expression via direct binding to *PPARA* promoter B (−49,076 to −49,064 upstream of the translation start site) [28] and observed that myocardial ERRα transcript expression is developmentally regulated in parallel with PPARα [61]. Interestingly, myocardial ERRα transcript expression is also up-regulated in animals fed high fat diets and in animal models of insulin-deficient DM (Huss, unpublished observation), providing further circumstantial evidence for a link between PPARα, ERR, and DM.

Our findings should be considered in the context of some potential limitations. Although genotype remained an independent predictor of 5-year mortality in both unadjusted and adjusted analyses, as well as in subgroup analyses performed in our largest racial group (Caucasians), replication in other studies is still warranted. Furthermore, our findings cannot be extrapolated to racial groups other than those represented in INFORM (Caucasians and African Americans) and should, therefore, be examined independently in other racial groups.

In conclusion, we have identified a *PPARA* promoter polymorphism that effects binding and activation of *PPARA* co-activators and is strongly associated with 5-year mortality in diabetic ACS patients. These findings suggest a genetic mechanism for the unfavorable cardiovascular outcomes in diabetic patients after ACS.

## Supporting Information

Table S1 PCR and Pyrosequencing primers and conditions.
(0.04 MB DOC)

Figure S1 Output of the web-based program PromoLign (http://polly.wustl.edu/promolign/main.html) [23], showing the 13 nucleotide variants identified within 10 kb of the transcription start site of *PPARA*.
(2.85 MB TIF)

Figure S2 Sequence comparison of *PPARA* −54,642 SNP and *PPARA* −54,645 SNP sites showing conservation of sequence between species (dbSNP build 126; http://genome.ucsc.edu/).
(2.04 MB TIF)
