# Supplementary material for: A PPARα Promoter Variant Impairs ERR-Dependent Transactivation and Decreases Mortality after Acute Coronary Ischemia in Patients with Diabetes
Source: PLoS One. 2010 Sep 3;5(9):e12584. doi: 10.1371/journal.pone.0012584 (PMC2933242; doi:10.1371/journal.pone.0012584)
Supplement: Table S1 — PCR and Pyrosequencing primers and conditions. (0.04 MB DOC) [file pone.0012584.s001.doc]

**Supplemental Table S1. PCR and Pyrosequencing primers and conditions.**

| **Polymorphism** | **(rs#)** | **Primer Sequence** | **Annealing**  **Temp (C°)** |
| --- | --- | --- | --- |
| *PPARA* |  | Forward 5’- TCCTGCAGGTTCTCAAGGTT -3’ |  |
| -54,642 G>A | (rs135561) | Reverse 5’- biotinTTCCATGCCAGCTCTCTTCT -3’ | 55° |
| (and -54,645 C>T) | (and rs135562) | Internal 5’- CAGGTTCTCAAGGTTGTA -3’ |  |
| *PPARA* |  | Forward 5’- CCAGGGGGAGGAAAGAGTGAA -3’ |  |
| -35,014 A>C | (rs135539) | Reverse 5’- biotinGCCACAACTAAGCAGGCAGTG -3’ | 62° |
|  |  | Internal 5’- GCAGAATTTAAATCCTAGGT -3’ |  |
