# Supplementary figures and images for: A PPARα Promoter Variant Impairs ERR-Dependent Transactivation and Decreases Mortality after Acute Coronary Ischemia in Patients with Diabetes
Source: PLoS One. 2010 Sep 3;5(9):e12584. doi: 10.1371/journal.pone.0012584 (PMC2933242; doi:10.1371/journal.pone.0012584)

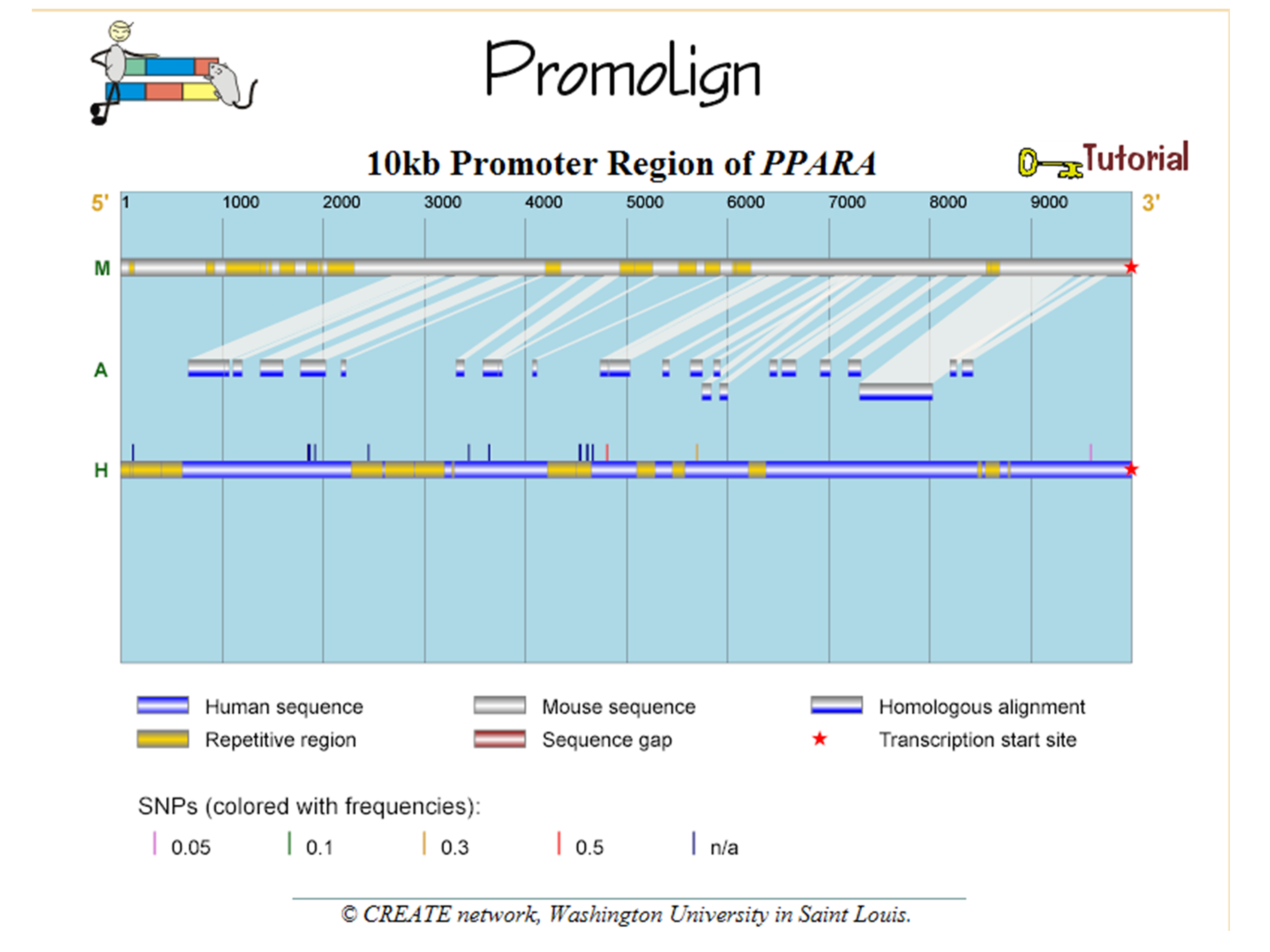

Supplement: Figure S1 — Output of the web-based program PromoLign (http://polly.wustl.edu/promolign/main.html) [23], showing the 13 nucleotide variants identified within 10 kb of the transcription start site of PPARA. (2.85 MB TIF) [file pone.0012584.s002.tif]

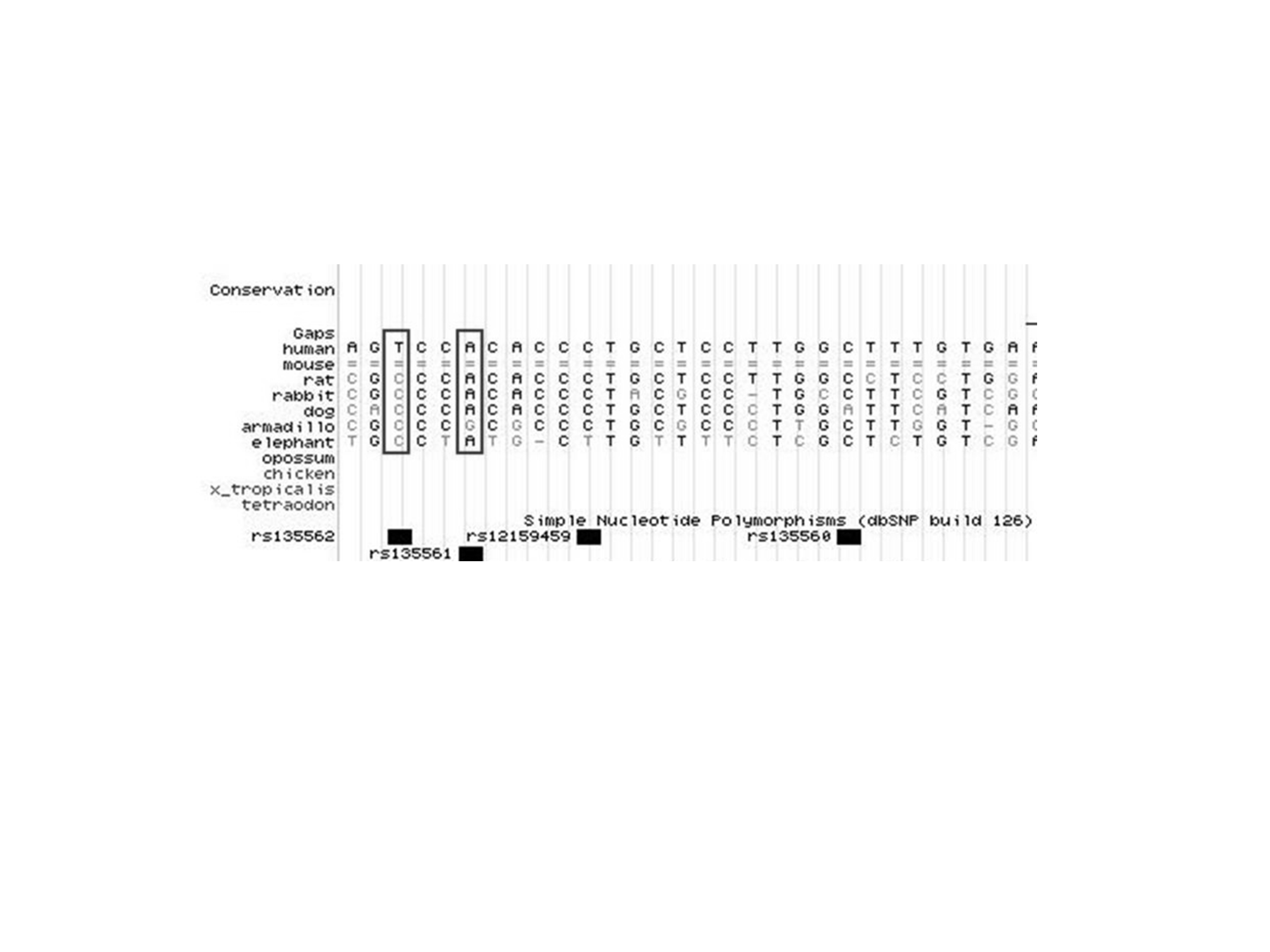

Supplement: Figure S2 — Sequence comparison of PPARA −54,642 SNP and PPARA −54,645 SNP sites showing conservation of sequence between species (dbSNP build 126; http://genome.ucsc.edu/). (2.04 MB TIF) [file pone.0012584.s003.tif]
